# Turning scientific serendipity into discoveries in breast cancer research and treatment: a tale of PhD students and a 50-year roaming tamoxifen team

**DOI:** 10.1007/s10549-021-06356-8

**Published:** 2021-08-16

**Authors:** V. Craig Jordan

**Affiliations:** Department of Breast Medical Oncology, The University of Texas MD Anderson Cancer Center, 1515 Holcombe Blvd., Unit 1354, Houston, TX 77030 USA

**Keywords:** Tamoxifen, Raloxifene, Selective estrogen receptor modulators, Women’s Health Initiative, Estrogen receptor

## Abstract

**Purpose:**

This retrospective, about a single “mobile” laboratory in six locations on two continents, is intended as a case study in discovery for trainees and junior faculty in the medical sciences. Your knowledge of your topic is necessary to expect the unexpected.

**Historical method:**

In 1972, there was no tamoxifen, only ICI 46, 474, a non-steroidal anti-estrogen with little chance of clinical development. No one would ever be foolish enough to predict that the medicine, 20 years later, would achieve legendary status as the first targeted treatment for breast cancer, and millions of women would benefit from long-term adjuvant tamoxifen therapy. The secret of tamoxifen’s success was a translational research strategy proposed in the mid 1970’s. This strategy was to treat only patients with estrogen receptor (ER)-positive breast cancer and deploy 5 or more years of adjuvant tamoxifen therapy to prevent recurrence. Additionally, tamoxifen prevented mammary cancer in animals. Could the medicine prevent breast cancer in women?

**Results:**

Tamoxifen and the failed breast cancer drug raloxifene became the first selective estrogen receptor modulators (SERMs): a new drug group, discovered at the University of Wisconsin, Comprehensive Cancer Center. Serendipity can play a fundamental role in discovery, but there must be a rigorous preparation for the investigator to appreciate the possibility of a pending discovery. This article follows the unanticipated discoveries when PhD students “get the wrong answer.” The secret of success of my six Tamoxifen Teams was their technical excellence to create models, to decipher mechanisms, that drove the development of new medicines.

**Summary of advances:**

Discoveries are listed that either changed women’s health or allowed an understanding of originally opaque mechanisms of action of potential therapies. These advances in women’s health were supported entirely by government-sponsored peer-reviewed funding and major philanthropy from the Lynn Sage Breast Cancer Foundation, the Avon Foundation, and the Susan G. Komen Breast Cancer Foundation. The resulting lives saved or extended, families aided in a time of crisis and the injection of billions of dollars into national economies by drug development, is proof of the value of Federal or philanthropic investment into unencumbered research aimed at saving millions of lives.

## Introduction

Jim Watson, in his book “Avoid Boring People,” writes “choose an objective apparently ahead of its time” [1]. In the 1960’s, when our journey begins, there were major advances in women’s health. The refinement of hormone replacement therapy (HRT) to treat menopausal symptoms and osteoporosis was an enormous success. The administration of conjugated equine estrogen (CEE) was recommended for women without a uterus and CEE with the addition of medroxyprogesterone acetate (MPA), to prevent endometrial cancer [2, 3], for post-menopausal women with a uterus.

The oral contraceptive, a mixture of a synthetic estrogen and a synthetic progestin, was pioneered at the Worcester Foundation for Experimental Biology (WFEB) under the directorship of Dr. Gregory Pincus. Dr. MC Chang conducted the biological evaluation of appropriate steroids, for the soon to be known “pill.” Dr. John Rock conducted the successful clinical trials [4]. The Food & Drug Administration (FDA) approved the oral contraceptive on June 23, 1960.

During the 1960’s, the application of combination cytotoxic chemotherapy was to revolutionize the treatment of childhood leukemia [5]. Cures became routine, when before, there was certain death. This advance was to be followed by the cure of Hodgkin’s disease [6]. Combination cytotoxic chemotherapy was viewed as the strategy to treat all cancers to achieve cures.

In the early years of the 20th Century, breast cancer was a disease with a relentless death rate. During the first half of the twentieth century, endocrine ablation (oophorectomy, adrenalectomy, or hypophysectomy) was routine for the treatment of metastatic (Stage IV) breast cancer [7]. In 1944, Haddow and coworkers [8] established, through a process of translational research, high-dose synthetic estrogen therapy as the first chemical treatment for any cancer. One in three patients responded to high-dose estrogen therapy if administered more than 5 years after menopause. However, during the inaugural Karnofsky lecture [9] at the annual meeting of the American Society of Clinical Oncology, Haddow lamented *“the extraordinary extent of tumour regression observed in perhaps 1% of post-menopausal cases (with oestrogen) has always been regarded as of major theoretical importance, and it is of some disappointment that so much of the underlying mechanism continues to elude us.”* In fact, Haddow [9] was not at all optimistic that cancer could be treated successfully; there were no tests for tumor-specific drugs to predict tumor vulnerability. This contrasted dramatically with the approach to treat bacterial infections using initial susceptibility testing in the laboratory.

My early involvement at the beginning of tamoxifen at ICI Pharmaceuticals division has recently been told [10, 11]. The triphenylethylene antiestrogen ICI 46,474 was discovered in the contraceptive program at ICI Pharmaceuticals by Drs Harper, Richardson, and Walpole [12–14]. The clinical development of tamoxifen was not a preplanned program by ICI Pharmaceuticals Division [10], but for me as a pharmacologist, it was “an objective ahead of its time.” We had a target in the tumor—the estrogen receptor (ER) [15–17].

Our Leeds Tamoxifen Team (1974–81) initially asked two straightforward questions:

1. What is the molecular mechanism of tamoxifen as an effective antiestrogenic breast cancer medicine?
2. If long-term adjuvant tamoxifen treatment is successful in patients, does acquired tamoxifen resistance occur in our model and are other tamoxifen derivatives superior to tamoxifen?

### The molecular mechanism of action of tamoxifen at the University of Leeds

In the case of tamoxifen, there was an unusual species specificity; tamoxifen was an estrogen in the mouse but anti-estrogenic in rats. The metabolites had been identified [18]; perhaps, tamoxifen was metabolized to estrogens in the mouse?

#### Clive Dix, Karen Porter (nēe Naylor), Linda Rowsby, and Graham Prestwich provide the foundation for tamoxifen action and application

Clive Dix was my first PhD student. He and Karen focused on our translational research plan to advance tamoxifen as an adjuvant therapy for breast cancer for patients with ER-positive breast cancer. The dimethylbenzanthracene (DMBA)-induced rat mammary carcinoma model would be investigated in full to formulate our clinical strategy. Early long-term tamoxifen treatment was discovered to be the correct strategy [19–23], and so it has proved to be in subsequent adjuvant clinical trials [24–27] following mastectomy.

At the University of Leeds, my Tamoxifen Team discovered that the tamoxifen metabolite, 4-hydroxytamoxifen, was not an estrogen but the first anti-estrogen with a high affinity for ER [28]. However, tamoxifen was not a classical prodrug but a weaker anti-estrogen [29]. 4-hydroxytamoxifen has rapid excretion and was never considered as a new therapeutic agent to treat breast cancer. However, 4-hydroxytamoxifen was active as an anticancer agent in the DMBA-induced rat mammary carcinoma model [30] but was too rapidly excreted to be considered for clinical use. 4-hydroxytamoxifen became the standard anti-estrogen used in vitro for cell culture studies of estrogen action in breast cancer. The mechanism of action of tamoxifen [31–33] and 4-hydroxytamoxifen [34–37] was studied extensively in the uterus of the immature rat. The model proposed was that 4-hydroxytamoxifen as an anti-estrogen, once bound to the ER, the ligand occupied the same area as the estradiol molecule [28]. But then opportunity, and a chance visit to the Wisconsin Clinical Cancer Center in Madison, created a break-through model that would decipher molecular mechanisms of tamoxifen as an antiestrogen and aid the clinical understanding of tamoxifen as an anti-cancer agent.

The advantage of an in vitro cell system is to study anti-estrogen action, without the complication of metabolism in the animal, which can create uncertainty for molecular pharmacology. In late 1978, I was invited to Wisconsin by Dr. Paul Carbone, the Director of the Wisconsin Clinical Cancer Center, to consider moving to Madison to establish a new Tamoxifen Team. What I had not anticipated was to meet Dr. Mara Lieberman who was working as a post-doctoral Fellow with Dr. Jack Gorski, the pioneer of ER cell biology [38], in the University of Wisconsin, Biochemistry Department. Mara shared with me her new studies, about to be published [39], on a novel system in vitro of dispersed immature rat anterior pituitary gland cells in culture, that responded to estrogen by synthesizing prolactin. In contrast, what I saw was an assay to study the structure–function relationships of nonsteroidal anti-estrogens in vitro*!*

### West to Wisconsin

**Fig. 1:**
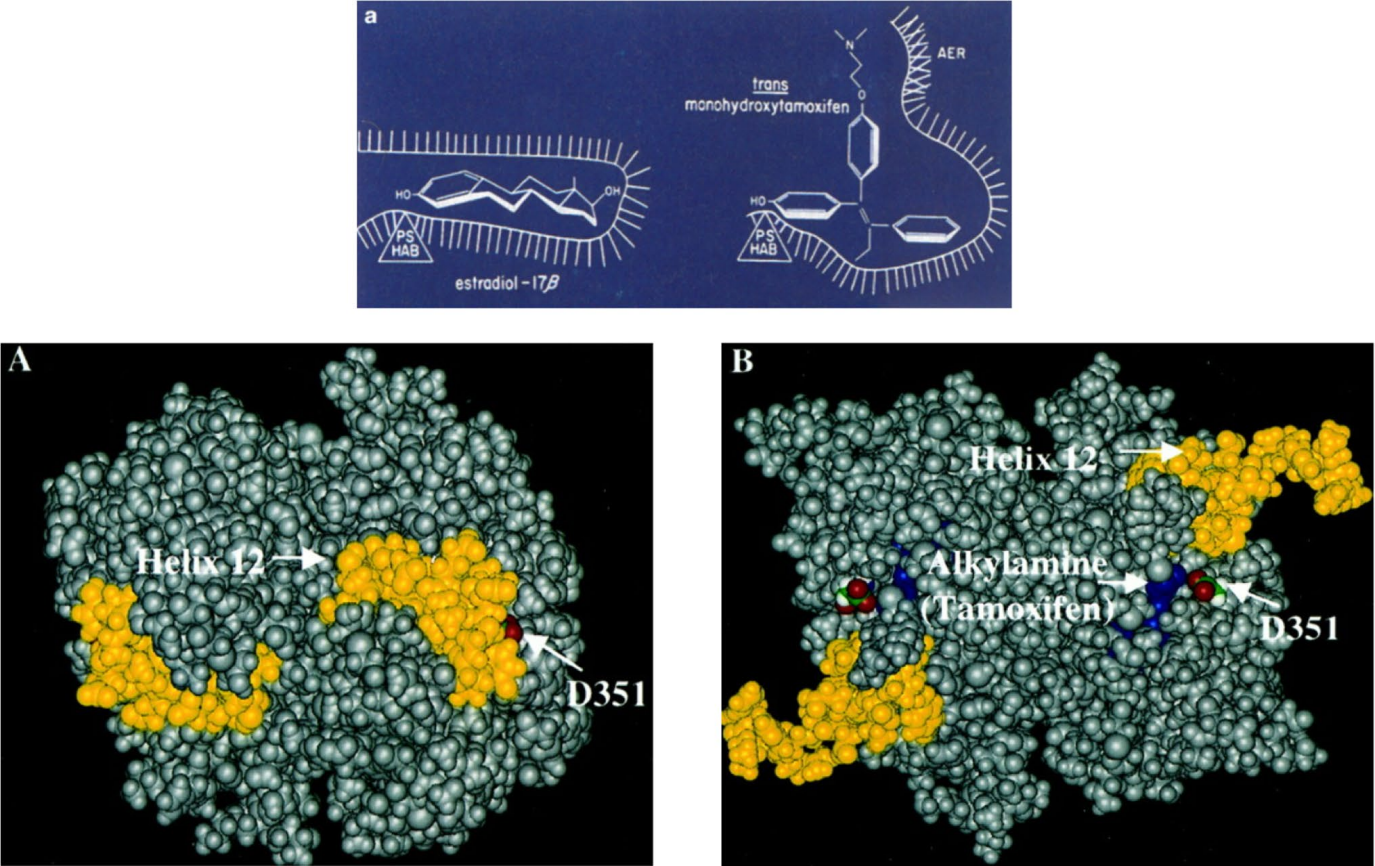
Top: The “crocodile model” of antiestrogen action of 4-hydroxytamoxifen [41, 48] and the side antiestrogenic chain interaction with the antiestrogenic region (AER) of the ER. Early work by Lednicer in the 1960s [161] concluded “that the presence of a basic group at *a given position in space* is required to obtain a molecule, which will antagonize the effects of concurrent estrogen administration.” The finding that the oxygen in the basic side chain can be replaced by a methylene group created compounds, which show the same potency as both antifertility agents and estrogen antagonists bolster the hypothesis. However, the introduction of ring methyl groups into triphenylethylene and triphenylethane antiestrogens *ortho* to the alkylaminoethoxy side chain is disadvantageous. This suggests that the side chain cannot be inhibited from rotation [162]. a from [41] and A, B from [106] with copy right permission

In 1980, I moved to the Department of Human Oncology in the Wisconsin Clinical Cancer Center to set up a new Tamoxifen Team. I hired Mara Lieberman and we rapidly published the first couple of papers [40, 41] that defined estrogen/anti-estrogen action at the ER. My ER model of anti-estrogen action with the dimethylaminoethoxy sidechain of 4-hydroxytamoxifen preventing closure of the ER complex by interacting with an “anti-estrogenic region” (AER) on the ER [41] (Fig. 1). We subsequently plotted out the predictable structure–function relationship of estrogens and anti-estrogens occupying the ligand-binding domain [42–47]. The results, using the prolactin assay, created structural rules whereby nonsteroidal compounds could be predicted to be agonists, partial agonists, or antagonists [43]. An antagonist could be predicted by extending the length of the strategically positioned anti-estrogenic side chain to engage the “anti-estrogenic region” in the ER [44]. The model became known as the “crocodile model”; an anti-estrogenic side chain was a stick in the jaws of the crocodile [48] (Fig. 1).

#### Anna Riegel makes discoveries with antibodies from the Jensen/Greene laboratory to support the “crocodile model” of estrogen/antiestrogen action

I wanted to deploy Elwood’s antibodies to determine whether Anna could identify differences between estrogens and antiestrogens once bound at the ligand-binding site of the ER.

The one enormous advantage we had was the first samples of tritiated 4-hydroxytamoxifen that we could use in vivo and in vitro [49, 50]. Overall, we concluded that if the goat antibody was preincubated with breast cancer cytosols, this dramatically reduced the affinity of estradiol for the ER. The antibody stopped the closing of the “jaws of the crocodile,” and [^3^H] estradiol “fell out” of the open ER. This did not occur if estradiol was prebound and locked after the jaws were closed. In contrast, [^3^H] 4-hydroxytamoxifen was unaffected by antibody treatment; the antiestrogen was wedged in the “jaws of the crocodile” [51]. Similar results were obtained using GH3 rat pituitary cancer cell [52]. Our molecular model was conceptually correct [28, 41] and now confirmed using an approach other than structure–function relationships.

First, we had to master new animal models to understand drug resistance to tamoxifen in human breast cancer cells. Two conclusions were to emerge: 1) Models to define mechanisms of relevance to understand and treat clinical breast cancer require analogous treatment durations used to treat clinical disease; years and not days of treatment in the laboratory are necessary. 2) The strategic goal became to decipher estrogen-regulated events that are essential to understand the molecular pharmacology of estrogen and anti-estrogen action in breast cancer during years of treatment.

#### Marco Gottardis and the study of acquired tamoxifen resistance in human breast cancer

At Wisconsin, Marco Gottardis, a PhD student, established an athymic animal model to grow estrogen-stimulated MCF-7 ER-positive breast tumors. Marco built numerous results from long-term experiments, one upon another. Tamoxifen treatment for months did not kill all tumor cells, as they could be resurrected into tumors by estrogen treatment even when tamoxifen treatment was given for up to 6 months [53]. It was noted by others that tamoxifen created a GI blockade [54, 55], that could be reversed by estrogen. But then a conceptual break-through occurred, which provided an invaluable insight into acquired resistance to tamoxifen in clinical breast cancer.

Osborne and colleagues [56] published that tamoxifen treatment of athymic mice for a year, resulted in the growth of MCF-7 tumors despite continuing tamoxifen treatment. Acquired resistance had occurred in an ER-positive tumor. Marco replicated these data, but asked two further questions:

1. If the resistant tumors are retransplanted into athymic mice and treated with vehicle, estrogen, or tamoxifen, are all tumors autonomous for growth?
2. If tamoxifen is an estrogen in mice, what occurs if tamoxifen-treated athymic rats are used? Tamoxifen is a weak estrogen but a potent anti-estrogen in rats.

The answer to the first question was that both tamoxifen or estrogen-treated tumors grew in athymic mice, but no tumors grew in the vehicle-treated mice. Tumors could utilize either estrogen or tamoxifen for growth [57]. The answer to the second question was that the acquired resistance to tamoxifen was no different in rats and mice [58]. Species-specific metabolism was unlikely.

The immediate advantage of Marco’s model was to test new “pure anti-estrogens” from companies seeking a second line therapy in the eventuality that tamoxifen-stimulated recurrence occurred in adjuvant tamoxifen-treated patients. Zeneca had a new “pure anti-estrogen” ICI 164,384 which performed well in Marco’s model [59]. As predicted, tamoxifen stimulated tumor growth, but ICI 164,384 did not cause the growth of tamoxifen-stimulated tumors and blocked estrogen-stimulated tumor growth. No treatment, i.e., no estrogen, as the mice were ovariectomized, so tumors did not grow.

A decade later AstraZeneca had two new anti-estrogenic agents faslodex (an injectable pure anti-estrogenic) and anastrozole (an aromatase inhibitor), for clinical testing following the failure of tamoxifen treatment. Both compounds performed well in an international clinical trial [60], thereby validating Marco’s Model for the study of new antiestrogen agents or no estrogen (an aromatase inhibitor) to treat tamoxifen-resistant breast cancer patients successfully.

Marco’s athymic mouse model of acquired resistance to tamoxifen was important to preserve, but we were unsuccessful in creating cell lines that replicated the fidelity of the animal model. There was no choice—we had to retransplant MCF-7 tumors biopsies into new generations of athymic mice for years.

#### A chance meeting: one experiment and a failed breast cancer drug keoxifene (raloxifene-to-be) creates Selective Estrogen Receptor Modulators (SERMs)! Serendipity!

This story has been told recently in full [61]. However, for the sake of illustrating the play of chance in discovery, it is a prime example of Serendipity. A visiting investigator at the University of Wisconsin, Dr. Urban Lindgren, from the Karolinska Institute in Sweden, asked me to create a model of osteoporosis for him in rats. If estrogen is good for building bone in ovariectomized rats, then perhaps an anti-estrogen would induce osteoporosis faster? Tamoxifen was the obvious choice, but I had several kilograms of a “failed” breast cancer drug keoxifene, that had little of tamoxifen’s estrogen-like activity.

I had found a publication from Baylor College of Medicine, in collaboration with the Space Center in Houston, that clomiphene would maintain bone in ovariectomized old breeder rats [62]. However, I knew that clomiphene was a mixture of isomers: enclomiphene (*trans*) and zuclomiphene (*cis*) which had anti-estrogenic or estrogenic properties, respectively. Perhaps, it was only the estrogenic isomer that built bone? I would use tamoxifen [14] and keoxifene [63] that were both pure compounds with anti-estrogenic properties in rats.

When Erik Phelps, a summer student, presented me with the results 3 months later, both anti-estrogens preserved bone density—it was a discovery! However, osteoporosis journals uniformly rejected the paper explaining “this makes no sense as estrogens preserve bone loss not anti-estrogens.” Finally, we published in Breast Cancer Research and Treatment, as the founding editor, Bill McGuire, shared my view of the novelty and translational potential of the findings [64].

We pursued translational research with tamoxifen (PI Richard Love, a clinician in our Department of Human Oncology) to determine the effect of tamoxifen on bone density in post-menopausal breast cancer patients. Dr. Love’s project was called the Wisconsin Tamoxifen Study. Meanwhile, other groups replicated the tamoxifen effects on bone in ovariectomized rats [65]. Eli Lilly, reinvented keoxifene as raloxifene to show the same [66], years after we [67], showed that tamoxifen was effective in maintaining bone density in post-menopausal patients.

Raloxifene was to be tested in post-menopausal women to prevent osteoporosis. HRT was known to increase breast cancer in women taking the combination of estrogen and progestins [68]. I proposed a different approach.

In 1990 [69], I had written down the roadmap for the pharmaceutical industry to develop the new group of medicines that we had discovered at Wisconsin now called SERMs. The roadmap was simply stated:

Is this the end of the possible applications for anti-estrogens? Certainly not. We have obtained valuable clinical information about the group of drugs that can be applied to other disease states. Research does not travel in straight lines and observations in one field of science often become major discoveries in another. Important clues have been garnered about the effect of tamoxifen on bone and lipids so it is possible that derivatives could find targeted applications to retard osteoporosis or atherosclerosis. The ubiquitous applications of novel compounds to prevent diseases associated with the progressive changes after menopause may, as a side effect, prevent breast cancer [69].

Now, with the results of Multiple Outcomes of Raloxifene Evaluation (MORE), we knew that it was possible to reduce the risk of breast cancer dramatically with a SERM [70] while treating postmenopausal women for osteoporosis. In fact, once raloxifene was on the market following FDA approvals. I calculated [71] that over a 10-year treatment period with raloxifene to prevent osteoporosis in the 500,000 women having prescriptions for raloxifene, that 27,230 breast cancers would be prevented. A noteworthy public health strategy had gone from the laboratory to the clinic. However, this advance of SERMs in medicine only occurred because of a chance meeting [61] and one experiment [64].

#### Doug Wolf, serendipity, and unanticipated discoveries

When Doug entered the Wisconsin Tamoxifen Team, I selected two major projects using either Marco’s model or creating his own model of acquired resistance to tamoxifen.

1. Growth factors were, at that time (late 1980s), a hot topic in breast cancer. Both Ethel Cormier (a PhD student) and Simon Robinson (a post-doctoral fellow) studied the role of growth factors to regulate tumor growth during tamoxifen therapy [72–75]. The growth factor, epidermal growth factor, reversed anti-estrogen anti-tumor action of tamoxifen in ER + breast cancer cells. Now, Doug took a different approach. If either tamoxifen or estradiol can stimulate the growth of Marco’s tamoxifen-resistance model, are the growth stimulants (estradiol or tamoxifen), that bind to the breast tumor ER, activating the same growth factor pathways?
2. In the late 1980s there were suggestions that the ER in breast cancer could harbor mutations. The evidence was weak, but does tamoxifen stimulate tumors to grow through a mutant ER?

#### Doug Wolf discovers a laboratory model to investigate estrogen-induced apoptosis: Serendipity!

Work on project 1 progressed slowly, despite the fact we had ready access to Marco’s model where both estradiol and tamoxifen stimulated MCF-7 tumor growth [57]. The design was simple: grow up tamoxifen-stimulated tumors until they were measurable and then randomize tumor-bearing mice into three groups: tamoxifen-treated, estradiol-treated, and injection of vehicle-treated as a control. Once tumors were easily measurable, animals would be sacrificed, and every known growth factor would be measured to determine the growth mechanism of tamoxifen or estradiol in tamoxifen resistance. A simple experiment with a unique animal model. However, there was no word from Doug, and he avoided contact with me for several months. Then suddenly, he came to my office and declared “You will have to write to the editor of Cancer Research to withdraw all of Marco’s papers. The work is not reproducible.” He had repeated the experiment twice with the same result. “Each time I have gone to measure the tumors grown up with tamoxifen, and now treated with low dose estradiol—all tumors had vanished.” I thought—it was a discovery!

The Marco Model tumors had been retransplanted for 5 years in tamoxifen-treated athymic mice. Haddow was speaking to us! In 1970, he stated in his Karnofsky lecture at the American Society of Clinical Oncology: *When the various reports were assembled at the end of that time it was fascinating to discover that rather general impression, not sufficiently strong from the relatively small numbers in any single group, became reinforced to the point of certainty; namely, the beneficial responses were three times more frequent in women over the age of 60 years than in those under that age; that estrogens may on the contrary accelerate the course of mammary cancer in younger women, and that their therapeutic use should be restricted to cases 5 years beyond the menopause* [9]*.*

The tumor required long-term estrogen-deprivation (LTED) to reconfigure tumor cell survival networks, but addition of estrogen “over stimulates” the breast cancer cell, resulting in death. Marco’s model had been retransplanted into tamoxifen-treated mice for more than 5 years. The anti-tumor action of estrogen was not a coincidence; it was a rule of ER-positive breast cancer biology.

It was 1992 and I had been invited to present a science lecture at the St. Gallen Breast Cancer Conference by the organizer, Professor Hans-Joerg Senn. This would be my talk: How tamoxifen causes long-term survival for breast cancer patients after the five years of tamoxifen is stopped [76]. Sparks flew at the meeting, when I suggested that a woman’s own estrogen kills prepared long-term tamoxifen-resistant micro-metastases, once 5 years of tamoxifen is stopped [76]. Suddenly physicians in the 1990’s did not like the idea of stopping adjuvant tamoxifen. My original battle cry in the 1980’s “tamoxifen forever!” had taken hold. However, what was Doug’s other discovery?

#### Doug Wolf and a search for a relevant mutation in the human breast tumor estrogen receptor

All that was known, in the late 1980’s, was the sequence of the human ER. However, that turned out to be somewhat controversial, as the original published sequence of the cloned human ER turned out to be harboring a valine for glycine point mutation at aa 400! [77].

Doug created his own stock of tamoxifen-stimulated tumors transplanted into athymic mice [78] and used the technique of polymerase chain reaction (PCR) single-strand conformational polymorphism (SSCP) to sequence the ER in new tumor lines. To our surprise, one of the MCF-7 tumor lines (MCF-7/MT2) contained ***large*** amounts of a mutant ER Asp351Tyr [79].

There was absolutely no evidence from the literature that this particular Asp351 amino acid played any role in anti-estrogen action: the actual crystallization of either 4-hydroxytamoxifen [80] or raloxifene [81] in the ligand-binding domain of the human ER was years in the future. We would work out the biological relevance of Asp351Tyr ER in anti-estrogen action, in my Tamoxifen Teams. But first we needed a new model to discover mechanisms. In this way, we would decipher the two chance discoveries by Doug Wolf: the antitumor action of low-dose estrogen following LTED in MCF-7 cells and a mutation Asp351Tyr in the ER of tamoxifen-stimulated breast cancer resistant to tamoxifen.

#### SY Jiang and the development of cellular models to define mechanisms in estrogen-deprived breast cancer

S. Y. Jiang was the first to transfect the ER cDNA stably into MDA-MB-231 ER-negative breast cancer cells (clone 10A) [82]. We discovered that estradiol now blocked the spontaneous growth of S30 MDA-MB-231 stable ER transfectants and anti-estrogens reversed that process.

In retrospect, the trick to achieve the success of stable transfection was for me to make the initial decision for S.Y. Jiang to clone all our cancer cell lines before we were up to speed with a molecular biology upgrade of my laboratory achieved by another PhD student, John Pink. John’s skill in molecular biology resulted in a new dimension for my Wisconsin Tamoxifen Team. We could now sequence ER in LTED cells [83, 84], clone out an ER-negative T47D cell line under LTED conditions [85, 86] and understand the regulations of ER synthesis in MCF-7 cells and T47D cells [87]. Under LTED conditions, MCF-7 cells expand their population of ER to survive. The cells are basically scavenging for estrogen and utilize the unoccupied ER to grow. In contrast, T47D:A18(C4:2) cells did not expand the ER population but lost the ER and became ER-negative [87].

However, the stably transfected ER-negative breast cancer cells S30 did not die with estrogen treatment! What if we created estrogen-deprived ER-positive MCF-7 cells and cloned out new cell lines? Unfortunately, nothing happened when the MCF-7:5C cells were treated with estrogen [88]. These MCF-7:5C cells and MCF-7:2A cells [83] were put in the freezer for the next decade! The aromatase inhibitors were advancing in clinical trials, so studying resistance to LTED in breast cancer would, some years in the future, be strategically important.

#### Mei-Huey Jeng and the pharmacology of synthetic progestins

Mei-Huey Jeng was a talented graduate student who continuously insisted she wanted to study the effects of progestins on the regulation of Transforming Growth Factor α (TGFα) and Transforming Growth Factor β (TGFβ) [1–3] in our breast cancer cell lines. After months of meetings I relented. Mei-Huey had made a discovery within three weeks [89]. Synthetic progestins that were 19-nortestosterone derivatives were estrogenic [90, 91]! Synthetic progestins, like medroxyprogesterone acetate (MPA), were not. Her other important contribution was the unexpected observation in S-30 stable transfectants, [82] that estrogen increased TGFα synthesis, but decreased TGFβ synthesis [92]. This seemed improbable as TGFα was a purported stimulant of cell replication, whereas TGFβ decreased cell replication. Nevertheless, an estrogen-stimulated gene marker TGFα was born for all subsequent studies, using stable transfectants in MBA-MB-231 cells to classify synthetic estrogens [93]. Her publication [90] using MPA would also, 20 years later, enhance the clinical understanding of Doug Wolf's initial observation [76] concerning the anti-breast cancer actions of low-dose estrogen therapy to induce apoptosis. We were unaware of how important this biology would become to decipher the results of the Women’s Health Initiative (WHI) trial [94].

#### Bill Catherino builds a new model to understand the molecular pharmacology of Asp351Tyr

Bill Catherino turned out to be a talented molecular/cellular biologist and created the BC-2 cell line of MDA-MB-231 10A stably transfected with the Asp351Tyr ER [95]. Bill’s PhD productivity in the laboratory was impressive [95–98]. He also classified a new synthetic progestin gestodene to be “estrogen-like” as it followed Mei-Huey’s earlier rule of being a 19 nor testosterone derivative [97].

In summary, we had made progress, by creating evidence, with multiple ER-negative breast cancer cell lines, transfected with specific mutations of the human ER that could enhance the estrogenicity for high-affinity anti-estrogens [82, 95, 99]. However, no clear molecular model had emerged of a mechanism, how the ER could be modulated predictably, to trigger breast cancer cell death, as demonstrated clinically by Haddow [8, 9].

### South to Northwestern at the Robert H. Lurie Cancer Center, Chicago

I was offered the position as Director of a new breast cancer program at the Robert H. Lurie Cancer Center in Chicago. The new Cancer Center Director was Dr. Steve Rosen, a talented clinician and enthusiastic medical scientist, with multiple philanthropic connections throughout Chicago, most notably the Lynn Sage Breast Cancer Foundation. I suggested that Dr. Monica Morrow, at the University of Chicago, would be an ideal Director for the proposed Lynn Sage Clinical Breast Cancer Program. Dr. Bill Gradishar, a medical oncologist, would complete our leadership triumvirate.

At the Robert H. Lurie Comprehensive Cancer Center, we created a Breast Program that went from nothing to world class in just 6 years. I was the PI of a Specialized Program in Research Excellence (SPORE) in Breast Cancer with Monica the co-PI. The visibility of our program was accelerated by a 3-day visit by Diana, the Princess of Wales, who was the primary speaker at the Breast Cancer Symposium I organized. There was talk of a return visit with her sons but then the tragedy of her death in Paris changed all that.

**Fig. 2:**
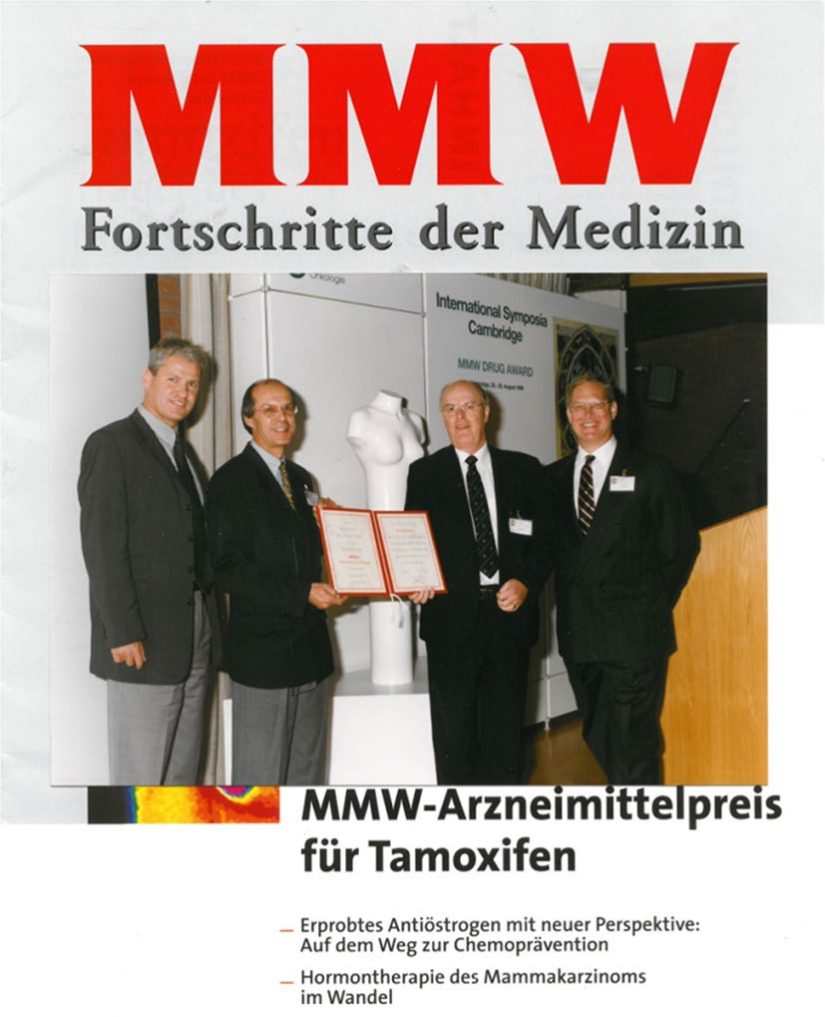
Tamoxifen recognized, August 1999, by the German MMW Drug Award. To celebrate this singular achievement for tamoxifen, Dr. Jordan was invited to organize and chair a three-day medical meeting for German physicians at the University of Cambridge, UK. The speakers in alphabetical order were M Baum, W. Eierman, B. Fisher, H.S. Füessl, W. Jonat, V.C. Jordan, M. Kaufman, and C.K. Osborne. Drs Ian Jackson (Zeneca) and V. Craig Jordan accepted the Award on behalf of AstraZeneca. By coincidence, on the day of his arrival in Cambridge, Dr. Jordan was contacted by the President of Northwestern University, Henry Binnen, to be told he was to be awarded the inaugural personal endowed chair: the Diana, Princess of Wales, Professor of Cancer Research, by Northwestern University

Within a few months of Diana’s death (I was actually on my way to Cambridge, to Chair a meeting to celebrate TAMOXIFEN being recognized by the German MMW Drug Award) (Fig. 2), I learned that Mrs. Anne Lurie, the benefactor of the Robert H. Lurie Comprehensive Cancer Center, had made a substantial donation to Northwestern University to create an endowed chair named: the Diana, Princess of Wales, Professor of Cancer Research. I was to be the inaugural recipient, and all this was officially approved by Diana’s blood family, the Spencer’s.

But what was going on in the laboratory that would sustain the academic trajectory of the Northwestern Tamoxifen Team for over a decade?

#### Research advances by the Northwestern tamoxifen team

The Northwestern Tamoxifen Team had fewer graduate students (Jennifer MacGregor, Rita Dardes, and Ruth O’Regan) than Wisconsin, but productivity was exceptional. Most importantly, there were excellent post-doctoral fellows who brought great innovation into the growing program. The Fellows included Anait Levenson, Debra Tonetti, Claudia Osipo, Zehan Chen, Katherine Pearce, Joan Lewis, and Eric Ariazi. Dr.Yuchi Iino, who spent a year in my laboratory at Wisconsin from Japan, was now sending his young surgeons (Drs Hiro Takei, Yasuo Hozumi, and J. Horoguchi) to Chicago to develop their research skills. Dr. Iino was elected as President of the Japanese Breast Cancer Surgeons Society and he highlighted our Tamoxifen Team. Surgeons also came to my laboratory from South Korea: Woo–Chan Park became Professor of Surgery at the Catholic University of South Korea in Seoul and Professor Eun–Sook Lee was appointed President, National Cancer Center, Seoul, S. Korea and Secretary General of the Asian National Cancer Centers Alliance. Tamoxifen Team (East) had a considerable influence on breast cancer research and treatment for two decades.

Finally, and most importantly, unlike all the other locations for the Tamoxifen Team, Northwestern had programs in the Departments of Surgery and Medical Oncology that allowed residents to spend a year (or 2) in my laboratory. This group of enthusiastic medical professionals was very important to create clinical relevance for our translational research.

#### Anait Levenson discovers the molecular pharmacology of how Asp351Tyr works to control estrogen/anti-estrogen action

I require every new member of the Tamoxifen Teams to spend their first few months in my laboratory writing a definitive review of the literature. At Northwestern, this was critical, as we were to rebuild an ER/anti-estrogen laboratory from empty laboratories that Steven Rosen constructed for my nascent Northwestern Tamoxifen Team. Anait (Ana) Levenson was my first recruit at Northwestern.

Our hot topic was our stable transfectants into MDA-MB-231 cells S-30, and BC-2, so I set Ana off to complete her first review on the transfection of ER (mainly transient) into ER-negative cancer cell lines [100]. She advanced rapidly to complete subsequent reviews on the MCF-7 cell line [101] and then on SERMs [102]. She was now fully up to speed on our topic at Northwestern.

**Fig. 3:**
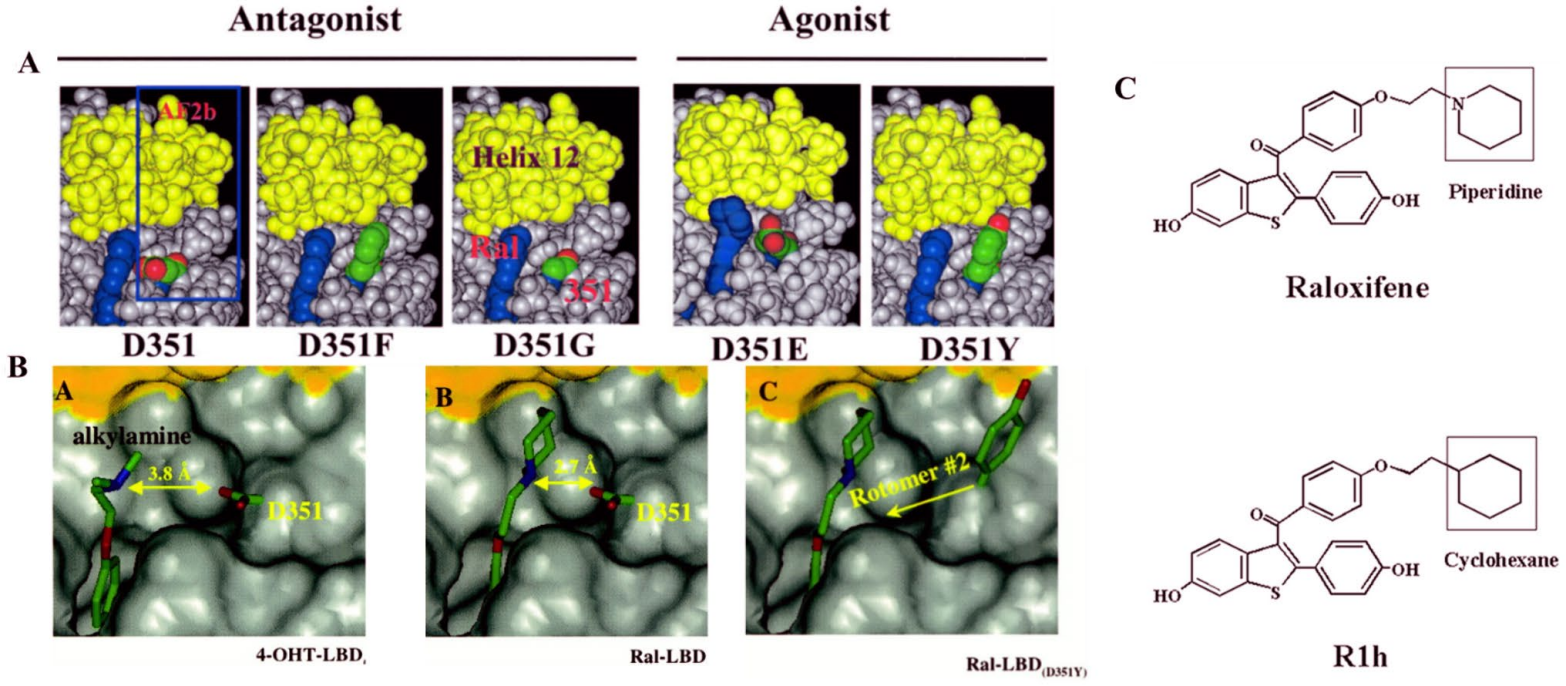
Prior to the resolution of the ligand-binding domain of the ER with either raloxifene [81] or 4-hydroxytamoxifen [80], we had identified amino acid Asp351 in the ER as the AER, i.e., the amino acid that interacts with the antiestrogenic side chain of raloxifene [103, 104]. This was followed by structure–function analyses of the ER complexes with mutations substituted at Asp351 (panels A). These were classified as retaining antiestrogenic properties or changing their actions to be estrogens (agonists). Panel 3A: Surface structures around amino acid 351 of raloxifene-bound LBDs of ERα. A structural model of dimeric human ERα bound to raloxifene was derived from the Protein Data Bank (code 1ERR) by removing all water molecules with the exception of the ordered water-forming H-bond with the O_3_ of raloxifene, adding hydrogens and minimizing in the consistent valence force field (CVFF) using Discover (Accelrys, San Diego, CA). Mutant receptors were constructed using Biopolymer (Accelrys) to replace Asp351 with Gly, Glu, Phe, or Tyr and to obtain a minimum energy rotomer for the mutant side chain. The results were visualized using Insight II (Accelrys). Molecular modeling of the surface structures of 4-hydroxytamoxifen-LBD (wild type) (**A**) or raloxifene-LBD (wild type) (**B**) and raloxifene-LBD (Asp351Tyr) (**C**). Asp351 replaced with Tyr351 in raloxifene-bound ERα LBD. To avoid steric clashes, Tyr351 is placed in a rotomer that projects the side chain upward. The side chain of Tyr351 is out of reach of the raloxifene side chain. Tyrosine residues typically lay down on the surface of proteins. In the ERR.pdb structure, small rearrangements in structure around Tyr351 are required to sterically accommodate the side chain. If this happens, the phenolic side chain would be oriented in rotomer #2. It is important to point out that the antiestrogenic N-containing side chain of tamoxifen (Fig. 3B) is further away from Asp351 than the N of raloxifene. This observation is consistent with the more estrogen-like actions of tamoxifen that results in higher blood clots and endometrial cancer than raloxifene [26, 70]; B, the piperidine side chain of raloxifene shields the charge of Asp351 and disturbs the local charge available for binding coactivators. As a result, AF1 and AF2 cannot collaborate properly, and TGF-α is silenced. C, the tyrosine at amino acid 351, changed the local charge available for coactivator binding because the piperidine can no longer shield the charge. Conformation of raloxifene-Asp351Tyr ERα to be 4-hydroxytamoxifen-ERα-like and TGFα gene is switched on. Panel C: Structures of raloxifene and the derivative R1h used in structure–function studies. Compound R1h is a raloxifene derivative that has a cyclohexane ring instead of a piperidine ring with no antiestrogenic actions. 3A from [107] and 3B from [106] with copy right permission

Ana deployed Shun-Yuan Jiang’s S-30 cells stably transfected with the wild-type ER gene and Bill Catherino’s BC-2 cells stably transfected with Asp351Tyr to determine whether keoxifene, a failed breast cancer drug with none of the estrogen-like properties of tamoxifen, would have a different molecular pharmacology via the Asp 351Tyr ER at a TGFα target. Yes, it did! Raloxifene was converted from an anti-estrogen to an estrogen (Fig. 3B). It was a discovery! It would appear that the anti-estrogenic side chain of keoxifene was interacting with the “anti-estrogenic region” [103] in a different way in the Asp351Tyr mutant obtained from an ER-positive breast cancer that had acquired estrogen-like resistance to tamoxifen. The anti-estrogenic side chain of raloxifene was not acting as “a stick in the jaws of the crocodile” if Asp was substituted by Tyr at aa 351. The “jaws of the crocodile” could now close with a tyrosine mutation of Asp351. We had proved experimentally that the area around aa 351 was the “antiestrogen region” (Fig. 3B).

The following year, an article was published in Nature (81) showing the X-ray crystallography of raloxifene (the new name for the failed breast cancer drug keoxifene) compared with estradiol in the ligand-binding domain of the human ER. All evidence now pointed to the fact that we had actually identified Asp351 as the predicted “anti-estrogen binding site” [41]. Cancer Research published our expanded molecular pharmacology mechanism for anti-estrogen action [104], integrating the newly published X-ray crystallography of raloxifene in the ligand-binding domain. We pursued the molecular pharmacology of the interaction of mutations of Asp351and a novel raloxifene derivative [105–107] (Fig. 3A & B). The raloxifene without a nitrogen-containing ring in the antiestrogenic side chain (Fig. 3C) was determined to be an estrogen. This provided the essential data proving the essential interaction of the side chain with the AER (Asp351) to create estrogen blockade. Through these structure–function studies, the individual answers to multiple questions created the fully functional mosaic we have today. Indeed, Asp351 is now recognized to be an essential anchor of the mutations in Tyr537Ser and Asp538Gly of helix 12, which is required to close the unoccupied ER creating acquired resistance to aromatase inhibitors [108]. Naturally, this has led to new investigations of ER blockers of the mutant ER [109, 110].

It is a strange twist of fate that Doug Wolf completed this work for his PhD with my Wisconsin Tamoxifen Team [79] to open the door to studies on Asp351, but then completed his post-doctoral Fellowship with Suzanne Fuqua in Texas and was part of the Team that discovered the mutant Tyr 537Ser in helix 12 of breast cancer patient material [111]. Doug’s contributions were important for the subsequent findings at both ends of the “closing the mutated ER story in AI resistance” a decade later [112].

#### Resurrection of Doug Wolf’s estrogen-induced apoptosis model in vivo

The influx of keen enthusiastic MDs-in-training provided a skilled group willing to work night and day on an enormous project to achieve a publication. This is illustrated by my decision to harness this “new force” in my Tamoxifen Team to repeat Doug Wolf’s earlier work [76] of the use of low-dose estrogen to treat acquired resistance to tamoxifen.

We established Doug’s model, [76] repeated it, and expanded it. The size of the tamoxifen-resistant tumors was the key; it was possible to cure animals with small tumors with low-dose estrogen. In contrast, animals with larger tumors would regress and regrow with estrogen. But most importantly, one could reintroduce tamoxifen and it was again an effective anti-cancer agent. There was ebb and flow of tamoxifen-resistant-and-responsive breast cancer cell populations.

These extensive new data were submitted to Clinical Cancer Research, but the referees were insistent on further animal studies. That was a problem! Most of the MD authors had returned to their clinical training or their home country. The savior of the situation was Dr. David Bentrem, a surgeon and new lab member. Within 4 months, he had completed all required experiments and our teamwork was accepted [113]. David completed numerous other studies in the laboratory, all of which were published in peer-reviewed journals [114, 115]. David is now Professor of Surgery at Northwestern University Hospital, Chicago.

#### Graduate students in the Northwestern Tamoxifen Team

Rita Dardes, the first Avon Scholar, focused on breast and endometrial cancer for her PhD thesis [116, 117]. Jennifer MacGregor (also an Avon Scholar) identified my laboratory for her PhD studies and proved herself as an exceptional scientist making discovery after discovery. She completed a review article to establish her educational database. Jennifer created an update of my initial Pharmacological Reviews article on nonsteroidal antiestrogens published in 1984 [118]. Her review was encyclopedic [119]. Jennifer covered a broad spectrum of studies on an Asp351Gly mutant to eliminate estrogen action of 4-hydroxytamoxifen in the ER complex [105], the development in vivo of acquired resistance to tamoxifen in the T47D:A18 breast cancer cell line [120], the correction of the classification by others of a new non-steroidal pure anti-estrogen when, in fact, it was a SERM! [121], and cross resistance to tamoxifen [122].

Ruth O’Regan focused on replicating clinical scenarios in our animal models that provided guidance for clinicians now that tamoxifen was not the only SERM, but raloxifene was now available as a medicine to treat osteoporosis and prevent breast cancer at the same time [70]. Drug cross-resistance between raloxifene and tamoxifen had never been addressed in the clinic since the first clinical trial of raloxifene that was unsuccessful in patients failing tamoxifen for the treatment of MBC [123]. Ruth provided clarity [124, 125] on the cross resistance of raloxifene in stimulating tamoxifen-resistant tumor growth. I was delighted to attend Ruth’s doctoral degree ceremony in 2000. She is now Chair in the Department of Medicine and Charles A. Dewey Professor of Medicine, University of Rochester, New York State!

Both tamoxifen and raloxifene would continue to catch headlines for two decades for the prevention of breast cancer in post-menopausal women. The prevention of breast cancer was a major clinical topic, having advanced from the initial animal study [126] to the successful Fisher trial funded by the NCI [127].

#### Joan Lewis: the discovery of an in vitro model and the mechanism for estrogen-induced apoptosis

We had been making good progress using our animal models of estrogen-induced apoptosis [128, 129] to confirm the published studies of Dick Santen’s group on the mechanism of estrogen-induced apoptosis in LTED breast cancer cells in culture [130]. It seemed that estrogen bound to the LTED MCF-7 ER then “something happened” to activate the extrinsic pathway, that caused cell death. Our tamoxifen [128] and raloxifene [129]-acquired resistance models in vivo both activated the extrinsic pathway, so everyone was on the same page. Except, what happened within the cancer cell after estradiol bound to the ER, which eventually activated the extrinsic pathway a week later?

**Fig. 4:**
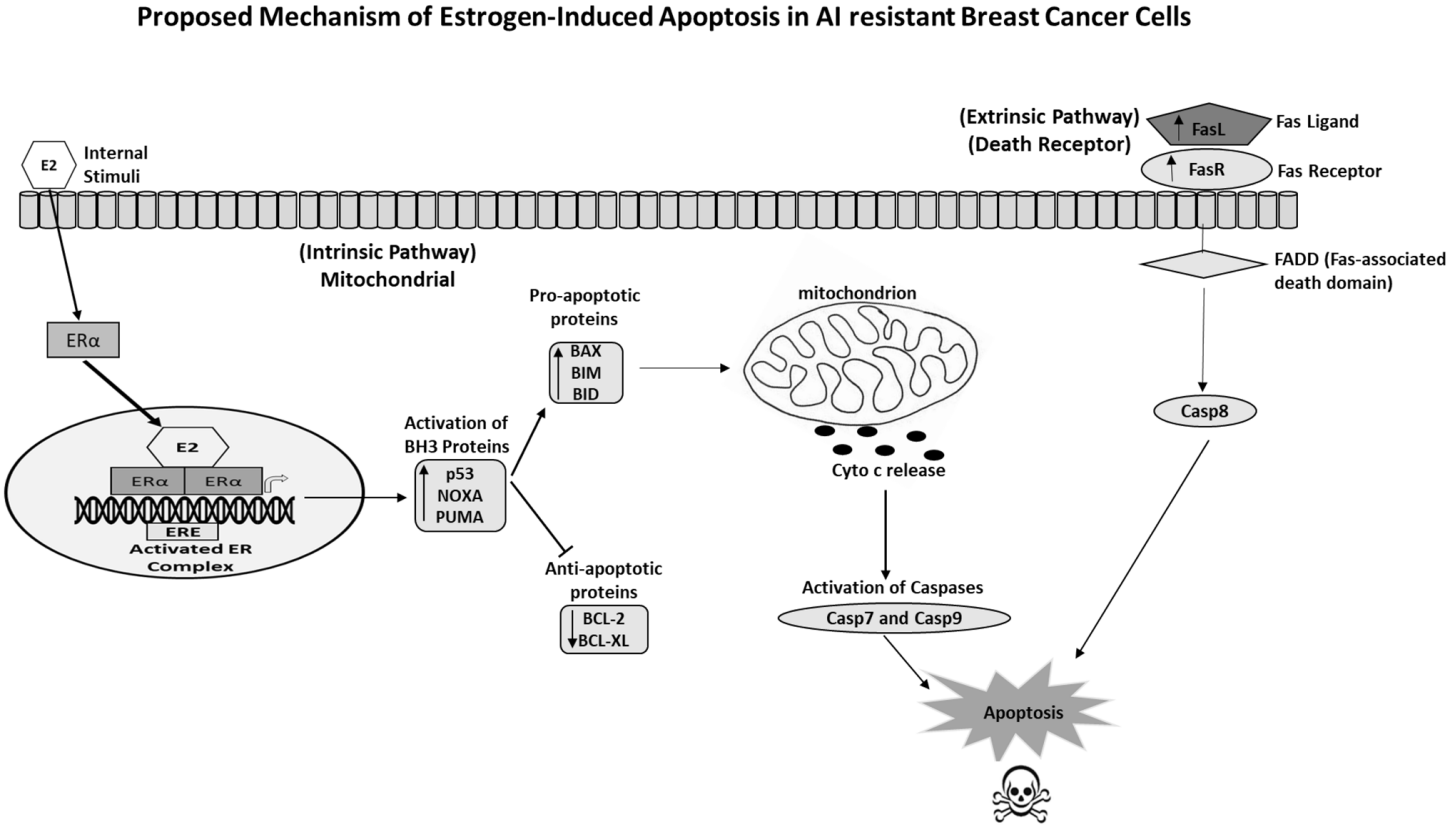
Joan Lewis mapped out [131, 132] the initial mitochondrial pathway (intrinsic that collaborates with the Death Receptor (extrinsic pathway) to cause apoptotic death). Ping Fan at Georgetown and MD Anderson completed the steps in apoptosis-modulating NF-κB activation by the endoplasmic reticulum stress sensor PERK [146] and the role of the glucocorticoid receptor to block estrogen-induced apoptosis. These molecular events in long-term estrogen-deprived breast cancer cells provide transparency to the results of the WHI: estrogen alone decreases breast cancer incidence, whereas estrogen plus MPA increases breast cancer incidence [148]

Joan’s project was to resurrect all Shun-Yuan’s MCF-7 cells [83, 88]. The MCF-7:5C and 2A cells were not responsive to E_2_ in culture. In contrast, in Joan’s studies, [131] all the MCF-7:5C cells died within a few days of low-dose estrogen treatment. This was odd, I thought, as Shun-Yuan’s publication was definitive—no apoptosis with estrogen [88]. I enquired whether Joan had repeated exactly, Shun-Yuan’s culture conditions she had published [88] a decade before. This is the first rule of reproducible science. Joan said “NO, I used the culture media that I used in my PhD to culture MCF-7 cells!” Her results were Serendipity! My reply was “Joan-never do that again and congratulations!” It was a discovery! Our cloned LTED ER-positive breast cancer model was used by Joan to map out both the intrinsic and extrinsic [132] steps of estrogen-induced apoptosis (Fig. 4). This allowed us to decipher the results of the Women’s Health Initiative that had now started to publish their breast cancer data [133, 134].

The MCF-7:2A cells are, again a lesson in experimental designs that do not reflect the realities of cancer biology. The cells were refractory to apoptosis over the standard laboratory culture time of 7 days [83]. However, an error in the laboratory that resulted in not *harvesting cells on time* (7 days) resulted in a discovery of apoptosis that was delayed until week 2. Lesson learnt: Laboratory assays must comply with the reality of cancer biology and not the convenience of PhD students!

### Forward to the Fox Chase Cancer Center (FCCC)

Nothing stays the same, everything changes. And so, it was with the successful Robert H. Lurie Comprehensive Cancer Center in 2005. The Director of the FCCC was Dr. Robert Young, a former Head of the Medicine Branch at the National Cancer Institute, and Dr. Robert Ozols, Deputy Director at Fox Chase. Both were committed leaders with a clear vision of how to maintain their Comprehensive Cancer Center Grant. Theirs was a great leadership team and Monica and I were delighted to be recruited to another great opportunity. They had an agenda and again we were in it!

It was time to rebuild a new Tamoxifen Team. I placed all my eggs in one basket and chose to focus our research efforts by obtaining funding to understand the mechanism of estrogen-induced apoptosis in breast cancer and explore clinical applications. I would apply for a Department of Defense Center of Excellence grant. This Federal program was master minded by Fran Visco creator and leader of the National Breast Cancer Coalition.

Joan Lewis and Eric Ariazi, members of the Northwestern Tamoxifen Team, both accepted positions at Fox Chase. Bob Ozols immediately ensured we would have a custom-made suite of interconnected laboratories for our growing Team. Most importantly, my executive secretary Marge Meenan would organize all the activities of our new Center of Excellence Grant: the only $12 million grant awarded nationally, that year, by the Department of Defense.

At Fox Chase, there were no immediate possibilities to train graduate students—or so I thought. I advertised in the scientific literature for post-doctoral Fellows, but then Marge informed me that Philipp Maximov M.D., on a Russian program from his medical school in Moscow, to train at the Fox Chase, wanted an appointment to see me. He stated that he could arrange with his university to sponsor a PhD with me. Welcome to the Fox Chase Cancer Center Tamoxifen Team, Philipp!

Joan Lewis became Philipp’s laboratory guide and mentor. This decision to recruit Philipp started a ten-year study of the molecular pharmacology of apoptosis in LTED cells to decipher the modulation of the precise trigger mechanism initiated by the ER ligand complex [135].

#### Eric Ariazi and our road map to understand the molecular events for estrogen-induced apoptosis in LTED breast cancer

Back at the FCCC, we needed a molecular road map for estrogen-induced apoptosis in breast cancer: Eric Ariazi was responsible for organizing, executing, and publishing that data base [136]. This massive project involved my whole Fox Chase Tamoxifen Team and collaborators from biotech. The whole project was my $12 million Center of Excellence Grant! He compared 3 cell lines MCF-7, MCF-7:5C, and MCF-7:2A over a 96-h period using gene arrays [136]. Eric developed a method called differential area under the curve analysis that identified genes uniquely regulated by E_2_ in 5C cells compared with both WS8 and 2A cells and hence were associated with estrogen-induced apoptosis. Estrogen signaling, endoplasmic reticulum stress, and inflammatory response genes were over expressed among the 5C-specific genes. Most importantly, the endoplasmic reticulum stress genes indicated that E_2_ inhibited protein folding, translation, and fatty acid synthesis. The endoplasmic reticular stress – apoptosis genes and caspase 4 were induced. Overall, this new roadmap of apoptosis triggered by estrogen [136] identified a mechanism through endoplasmic reticular stress and inflammatory responses in LTED breast cancer.

Our biological data base [76, 78, 79, 105–107, 113, 128, 129] now set the stage to decipher the published results for the WHI [133, 134]. When these clinical data were first reported, there were articles in the New York Times that there was no ready explanation for estrogen (CEE) alone to prevent increases in breast cancer incidence. We and others had already contributed a number of refereed publications to the literature [130 and see above]. The role of estrogen-induced apoptosis in LTED breast cancer (which was the situation in the WHI as there was at least a 5-year gap built in the study design after menopause) was clear. That being said, there was no mechanism defined to explain how MPA reversed the anti-breast cancer effects of CEE alone and actually increase breast cancer incidence over no treatment controls.

### Going to Georgetown

Nothing stays the same, everything changes. Dr. Morrow took a position as Chief, Breast Service, Department of Surgery, in a beautiful new building at Memorial Sloan Kettering, New York. I accepted the position as Scientific Director, at the Georgetown/Lombardi Comprehensive Cancer Center and I held the Vincent T. Lombardi Chair of Translational Cancer Research. At Georgetown, we again occupied custom-built interconnected laboratories.

The new Georgetown Tamoxifen Team sprang up quickly with Ping Fan as a Research Assistant Professor, who had come to my laboratory from Dick Santen’s laboratory and Surojeet Sengupta, who had joined my laboratory from Benita Katzenellenbogen’s laboratory. Not only did I take the remaining years of my Center of Excellence Grant to Georgetown, but the Susan G. Komen Foundation selected me as a Komen Scholar. Their investment in my Tamoxifen Team allowed me to invite Phillip Maximov back from Moscow to be an International Susan G. Komen post-doctoral Fellow at Georgetown. We planned to investigate the molecular pharmacology of estrogen-induced apoptosis in breast cancer. An added bonus at Georgetown was that the Director of the Cancer Center Education Program was my former PhD student from Wisconsin, Anna T. Riegel (she is currently the Senior Associate Dean for Biomedical Graduate Education and the Celia Rudman Fisher Professor of Oncology and Pharmacology).

#### Ping Fan targets c-Src to enhance estrogen-induced apoptosis and makes an unanticipated discovery

A principal goal of our research into endocrine-resistant breast cancer was to improve tumor responsiveness to estrogen and expand the effectiveness of estrogen-induced apoptosis. To this end, a c-Src inhibitor 4-amino-5-o-phenyl(4-chlorophenyl)-7(t-butyl)pyrazolo[3,4-d] pyrimidene (PP2) was employed to block signaling pathways. cSrc is present in 80% of breast cancers, so we reasoned it was important for breast cancers to survive.

Results demonstrated that PP2 blocks ER-negative cells growth but had little effect on ER-positive cells. Surprisingly, PP2 blocked estrogen-induced apoptosis and restored estrogen-stimulated growth, in LTED breast cancer cells [137]. This was exactly the opposite of our anticipated results! Culture times, used to study cell population changes with MCF-7:5C cells, were clinically relevant (8 weeks), using E_2_ plus PP2, E_2_ or PP2 alone. The cells that grew with E_2_ plus PP2 were identified as MCF-PF cells, with enhanced IGF-IRβ, that increased activity of Akt, and blocked apoptosis [138]. Further study demonstrated that RNA-interference of c-Src or PP2 blocked apoptosis. In fact, PP2 inhibition of c-Src not only blocked estrogen-induced apoptosis but also restored estrogen-stimulated growth in LTED MCF-75C cells [139]. An extensive study of MCF7:PF cells led to the discovery that [139] she had recapitulated in vitro the “Marco Model” of tamoxifen/estrogen-stimulated MCF-7 cells in vivo in tamoxifen-treated athymic mice over 1–2 years [57, 58]. Ping’s MCF-7:PF cells now provided precise mechanisms of action and were used to complete a comparative efficacy study of a whole range of available SERMs that might have been evaluated and failed as second line agents following acquired tamoxifen resistance [139].

Here again was an unanticipated laboratory result that was not the goal of the study. Serendipity led to discovery.

#### Deciphering the WHI trials: graduate students and progress in modulating estrogen-induced apoptosis in breast cancer

My surprise at Georgetown was that two graduate students Ifeyinwa (Ify) Obiorah and Elizabeth Sweeney both turned up in my laboratory and announced they wished to do their PhD with me as they had no interest in doing rotations-Deal!

Ify was fortunate to have Angela Brodie (the Mother of aromatase inhibitors) [140] and Anna Reigel on her committee. Her laboratory skills were excellent, and she started by addressing multiple inter-related projects of clinical significance: the efficacy of CEE on estrogen-induced apoptosis in breast cancer [141], and numerous studies on the structure–function relationships of estrogenic ligands that bind the ER [142]. I recall Ify’s first annual review of progress, when Anna Riegel noted that “these effects, you are documenting on estrogen-induced apoptosis are very odd. Why does this process with estrogen take so long when chemotherapy kills in 24 h?” [143]. This important observation refocused our work on time to apoptosis and documentation of the chain of events for estrogen-induced apoptosis with planar and angular estrogens. However, we were aware that a compound like bisphenol was not simply “a slow acting estrogen to induced apoptosis.” [144]. An earlier PhD student Philipp Maximov had demonstrated that the bisphenol ER complex was initially able to block estrogen-induced apoptosis—the bisphenol complex with ER was an antiestrogenic complex [135]. The molecular mechanism would be solved five years later.

Elizabeth Sweeney made a discovery that advanced our understanding of the WHI. The WHI was initiated to establish whether women benefited overall for CCE + MPA or CEE alone with an improvement in their health. After 20 years of a clinical trial and extensive observation of women post-therapy, the question was answered: there was no health benefit overall nor was there a reduction is coronary heart disease which was the goal of the study.

However, in the early years of the 21st Century, the mechanism to be resolved was why CEE alone causes a decrease in the incidence of breast cancer, whereas addition of MPA resulted in the expected increase in breast cancer.

Long-term estrogen deprivation was the key. Examination of the WHI protocol revealed that patients were being treated with CEE or MPA + CEE on average 5–10 years after menopause. Estrogen (CEE alone) was killing nascent breast cancer cells in well women. But why did MPA, a synthetic progestin, that we knew did not affect the growth of MCF-7 breast cancer cells [89], reverse estrogen-induced apoptosis?

The closing sentence of Eric Ariazi’s PNAS paper [136] stuck in my mind: “Furthermore, these findings lead to the hypothesis that anti-inflammatory agents prescribed for ancillary clinical problems should not be used during anti-tumor estrogen therapy.” The other part of the solution was that I remembered being taught, as an undergraduate pharmacologist, that MPA has glucocorticoid activity and that was why women experienced weight gain during high-dose treatment for breast cancer.

Elizabeth Sweeney and Ping Fan worked diligently to demonstrate that MPA or dexamethasone could block estrogen-induced apoptosis in MCF-75C cells. The paper [145] attracted much attention in academic circles by being selected for Faculty 1000 Prime. However, we still had to understand precisely the molecular events involved in the modulations of estrogen-induced apoptosis and the molecular role of glucocorticoid action.

But now it was time for us to make our last move to the MD Anderson Cancer Center.

### “If I was to offer to buy your brain, how much would that cost?”

The above were the words presenting me with a job offer at the MD Anderson Cancer Center. This was at a cocktail party at the AACR annual meeting! The MD Anderson was to be my final Tamoxifen Team.

Drs. Ping Fan and Philipp Maximov were up to the challenge of coming to Houston. As luck would have it, Dr. Balkees Abderrahman was on a 2-month medical educational visit to MD Anderson, and she impressed me with her writing and analytical skills. She was hired as a post-doctoral Fellow because of her recent MD degree but it was clear she had talent, grasping complex scientific problems in estrogen-induced apoptosis. There was no possibility that she could return to student life as a PhD student to complete a PhD in Houston that would take over 5 years. However, the University of Leeds came to the rescue. She entered a “split-site” program “for applicants of very high quality” between Leeds, the degree granting institution, and MD Anderson where she completed her thesis work in less than 3 years, as a member of my MD Anderson Tamoxifen Team.

Three lingering scientific questions remained to be resolved at MD Anderson:

#### Ping Fan completes our molecular mechanism to explain the increase in breast cancers in the CEE/MPA-treated women ten years after menopause in the WHI

Over the previous 15 years, we had assembled the mosaic of laboratory models that mimic LTED breast cancer in vivo and in vitro. NFκB was depleted during estrogen-induced breast cancer regression in vivo [128, 129]. Models in vitro indicated that estrogen-induced apoptosis was heralded by a massive increase in subcellular inflammation [136]. Ping and graduate student Lizzie Sweeny, at Georgetown, had demonstrated that the glucocorticoid properties of MPA blocked estrogen-induced apoptosis [145].

Ping’s study at MD Anderson identified the molecular link with the activation of NFkB [146] and the suppression of apoptosis with glucocorticoids and MPA [147]. The resulting publications at MD Anderson [148] created a scientific solution for the breast cancer results of the WHI based on experimental evidence.

#### Balkees Abderrahman, Philipp Maximov, and Ramona Curpan define the molecular mechanism of action of the partial estrogen agonist bisphenol to delay apoptosis

We were fortunate to have published structure–function relationship studies of triphenylethylenes that we were custom synthesized at the Fox Chase Cancer Center [149]. Philipp Maximov, Balkees Abderrahman, and our molecular modeler Ramona Curpan in Romania worked as a team to solve the molecular mechanism of how bisphenol delays apoptosis for a week [150–152].

Ramona Curpan used molecular dynamics computer simulations to demonstrate that not only does the phenolic hydroxyl of bisphenol in the stilbene structure bind in the position in the ER that is naturally occupied by the 3 phenolic hydroxyl of estradiol, but also the second phenolic hydroxyl of the triphenylethylene of bisphenol, now interacted with Thr347 to displace Asp351. This key Asp351 must be precisely positioned as it is essential to bind amino acids in helix 12 to seal over a planar estrogen-like estradiol and activate the ER complex [150, 152]. It is the side chains of 4-hydroxytamoxifen and raloxifene that shield Asp351 to prevent this closure like a “stick in the jaws of a crocodile” [48]. It is also important to note that raloxifene shields [152, 153] Asp351 more effectively than 4-hydroxytamoxifen (Fig. 3). This explains why raloxifene is the more complete anti-estrogen in the uterus of mammals, whereas 4-hydroxytamoxifen has more estrogen-like activity in the uterus. This is reflected by more endometrial cancer with tamoxifen [26, 127] but none with raloxifene [70].

The bisphenol ER complex prevents an initial response to E_2_ to trigger apoptosis. The bisphenol complex is, in fact, an anti-estrogenic influence initially [150] displaces the Asp351 to prevent the initial full activation of the ER complex. Nevertheless, the bisphenol ER complex eventually generates enough UPR to trigger delayed apoptosis.

#### Balkees Abderrahman and Ramona Curpan define the molecular mechanism of action of the estrogen mimic TTC-352.

The answer to the third question has its roots in an independent research effort by former members of the Northwestern Tamoxifen Team. Dr. Debra Tonetti went to the University of Illinois campus in Chicago. She and Dr. Greg Thatcher created a novel group of medicine they called Selective Estrogen Mimics—that evolved into ShERPAs (Selective human Estrogen Receptor Partial Agonists).

Compounds were synthesized [154] and a few selected for further study. Former Tamoxifen Team member, Ruth O’Regan, was then at the University of Wisconsin, Carbone Comprehensive Cancer Center and Division Chief Hematology, Oncology Palliative Care, Department of Medicine, where she headed the clinical team to test TTC-352, as a new promising estrogen-like treatment for endocrine-resistant MBC [155]. It is important to note that Ruth was a coauthor on the breakthrough article by Yao et al. 2000 [113] on the potential clinical application of low-dose estrogen therapy. The body of laboratory work from the Northwestern Tamoxifen Team was confirmed in patients, half a decade later, by Dr. Mat Ellis [156] using high- and low-dose estrogen to provide clinical benefit for breast cancer patients who recurred during aromatase inhibitor treatment.

Dr Abderrahman conducted experiments with two ShERPAS BMI-135 [152] and TTC-352 [151]. The ShERPAs were compared and contrasting with estetrol, a fetal metabolite of estradiol also being tested in clinical trial for the treatment of antihormone-resistant breast cancer [157].

Both BMI-135 and TTC-352 were weak full agonists, whereas bisphenol was a true partial agonist. The journey from the laboratory to successful clinical testing with mechanisms deciphered was complete. Balkees Abderrahman, MD, PhD, during her tenure on the MD Anderson Tamoxifen Team as a fellow and PhD student at Leeds University was selected as a Forbes 30 under 30 in Science and selected to attend the 2021 Meeting of Nobel Laureates in Lindau, Germany.

#### What was achieved during the 50-year tenure of the Tamoxifen Team?

**Fig. 5:**
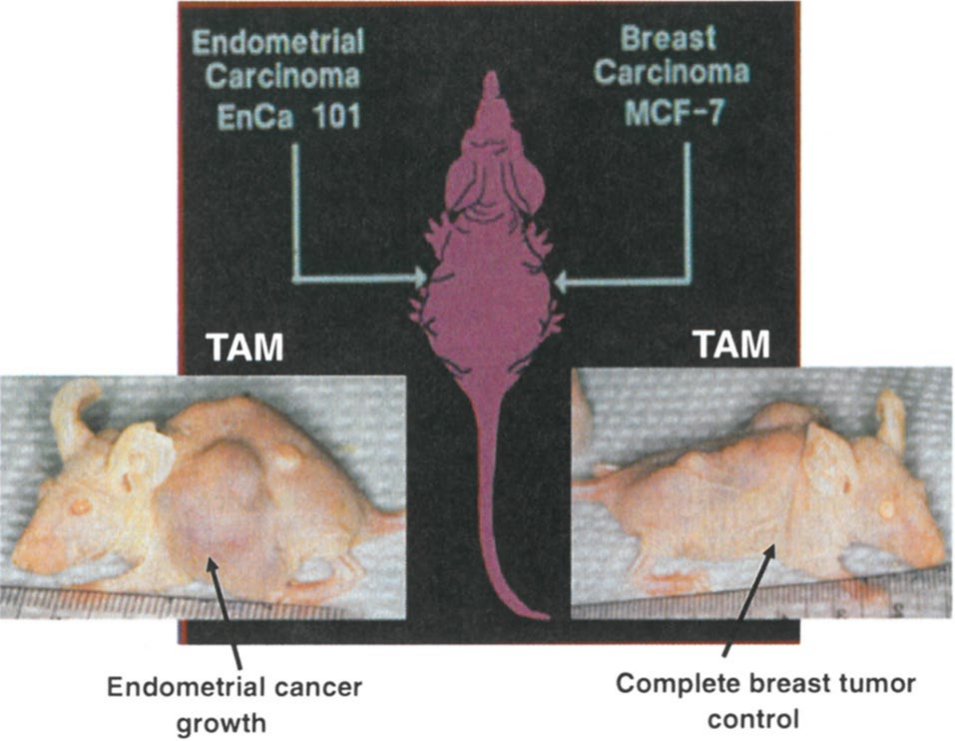
The pioneering bitransplantation study by Gottardis [158] with an ER-positive breast tumor (MCF-7) implanted in one axilla and an ER-positive endometrial tumor (EnCa 101) in the other axilla. Tamoxifen blocks estrogen-stimulated growth of the breast tumor, but tamoxifen encourages the growth of the endometrial tumor

During the 1970s, translational research strategies were created by the first Tamoxifen Team at the University of Leeds that changed medicine. This was not the priority of ICI Pharmaceuticals Division at Alderly Park, as their focus was to achieve approval for tamoxifen worldwide, as a safe effective medicine to treat stage IV breast cancer. Ours was an independent commitment by the Tamoxifen Teams. The success of the “roving Tamoxifen Team” can be summarized:

1. The proposed use of tamoxifen for the long-term adjuvant treatment of node-positive ER-positive breast cancer, and prevention in high-risk women, was successful in translation to clinical trials and FDA approvals [10].
2. Concerns about the risks of endometrial cancer raised by translational research at the University of Wisconsin [158] were examined (Fig. 5) and safety measures were introduced.
3. Study of the target site specificity of tamoxifen and raloxifene (a failed breast cancer drug initially called keoxifene) resulted in the creation of a new group of medicines proposed at the University of Wisconsin. The discovery of Selective Estrogen Receptor Modulation [64, 69, 159, 160].
4. The discoveries of a) estrogen-induced anticancer action of low-dose estrogen, and b) the elucidation of the molecular pathway for estrogen-induced apoptosis [76, 113, 129, 132, 146].
5. Translational research by past tamoxifen team members to aid in the development of new weak estrogenic molecules for the treatment of LTED breast cancer [151, 152, 155].
6. Deciphering the molecular mechanism of action of the WHI trials on lowering the incidence of breast cancer with CEE and elevating the risk with CEE plus MPA [145–147].

**Fig. 6:**
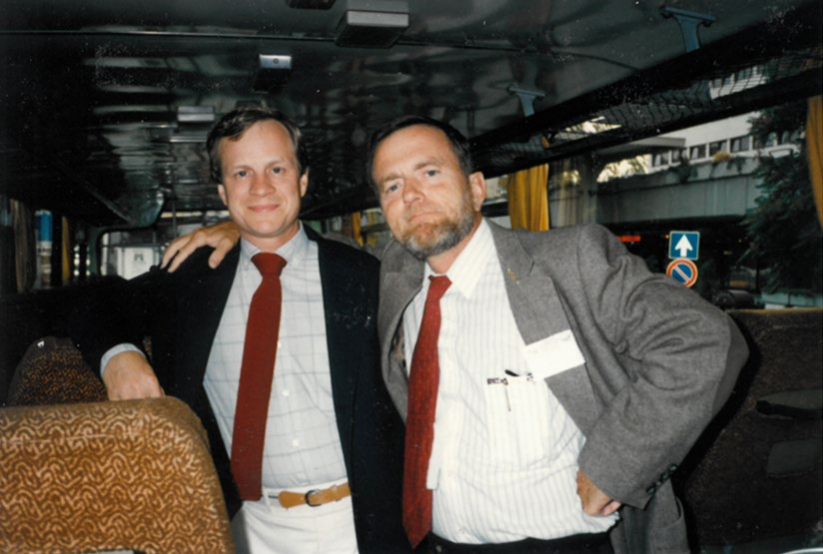
Dedication to the late Bill McGuire (right). He and Geoff Greene, of the University of Chicago (left), are getting onto a bus for participants in an International Cancer Congress in Budapest, Hungary, in the mid 1980s

## Dedication

On March 25, 1992, my friend and mentor Bill McGuire (Fig. 6) died. After his death, I received a letter he had written (this was before emails became popular as formal business communications!) inviting me to be guest editor for a special issue of *Breast Cancer Research and Treatment* (Jordan, 1994, BCRT, vol 31 #1). As you can imagine, opening his letter after his death, during a diving holiday in Mexico, was a unique experience! At the time of his death, circumstances did not permit me to attend his memorial service. I have used this opportunity to pay tribute to Bill and the twenty years of his friendship and guidance. He always gave me sound advice and challenged my resolve. One occasion stands out. I was invited to be a speaker at a symposium as part of an AACR cancer research meeting in Atlanta. The end of my presentation came, and I considered it only appropriate to thank my mentors Drs. Bill McGuire, Elwood Jensen, and Jack Gorski. Bill’s response was unexpected: “Never do that again! I am sent many applications of yours for awards or papers to review. That will stop if it is known that you consider me as your mentor” Wise words!
